# When the interoceptive and conceptual clash: The case of oppositional phenomenal self-modelling in Tourette syndrome

**DOI:** 10.3758/s13415-024-01189-6

**Published:** 2024-05-22

**Authors:** D. Parvizi-Wayne, L. Severs

**Affiliations:** 1 Department of Psychology, Royal Holloway University of London, London, UK; 2 Centre for the Philosophy of Science, Faculty of Sciences, University of Lisbon, Lisbon, Portugal; 3 Ruhr-Universität Bochum, Institute of Philosophy II, Bochum, Germany

**Keywords:** Active inference, Embodied cognition, Free energy principle, Tourette's, Mental health, Development

## Abstract

Tourette syndrome (TS) has been associated with a rich set of symptoms that are said to be *uncomfortable, unwilled,* and *effortful* to manage. Furthermore, tics, the canonical characteristic of TS, are multifaceted, and their onset and maintenance is complex. A formal account that integrates these features of TS symptomatology within a plausible theoretical framework is currently absent from the field. In this paper, we assess the explanatory power of hierarchical generative modelling in accounting for TS symptomatology from the perspective of active inference. We propose a fourfold analysis of sensory, motor, and cognitive phenomena associated with TS. In Section 1, we characterise tics as a form of action aimed at *sensory attenuation*. In Section 2, we introduce the notion of *epistemic ticcing* and describe such behaviour as the search for evidence that there is an agent (i.e., self) at the heart of the generative hierarchy. In Section 3, we characterise both epistemic (sensation-free) and nonepistemic (sensational) tics as habitual behaviour. Finally, in Section 4, we propose that ticcing behaviour involves an inevitable conflict between distinguishable aspects of selfhood; namely, between the minimal phenomenal sense of self—which is putatively underwritten by interoceptive inference—and the explicit preferences that constitute the individual’s conceptual sense of self. In sum, we aim to provide an empirically informed analysis of TS symptomatology under active inference, revealing a continuity between covert and overt features of the condition.

## Introduction

Tourette syndrome (TS) is a neurodevelopmental disorder diagnosed on the basis of multiple chronic motor and vocal tics (Johnson et al., 2023; Leckman, 2002). Despite considerable advances in our understanding of the pathophysiological basis of TS, the condition has resisted explanation in terms of either its neurological and psychiatric aetiology, and many factors are known to contribute to the unique expression and symptomatology of TS. Therefore, the goal of this paper is not to attempt to provide a unified explanation of its genesis, nor of its expression and maintenance. Nevertheless, consistent with naturalistic approaches to phenomenology, we aim to provide an initial account of the sensory, cognitive, and motor symptoms of TS via a neurocomputational perspective. Indeed, this account accommodates many of the qualitative aspects of TS symptomatology and attempts to systematically link the phenomenological level of description and empirical level of description in TS such that both levels mutually constrain one another in a productive manner (Lutz, 2002; Ramstead et al., 2022; Smith et al., 2022; Varela, 1997). More specifically, we assess the role of hierarchical generative (self) modelling in TS, from the perspective of active inference—according to which organisms take actions that change subsequent sensory input in order to realise the predictions generated by a model of the kind of thing it is (Friston, 2009, 2010, 2019; Ramstead et al., 2023a).

Despite a careful focus on the phenomenology of TS and its role in diagnosis, treatment, and phenotyping, there is still a relative paucity of formal approaches to link the symptomatology of TS to underlying constructs in computational neuroscience and biology (Grados & Mathews, 2009; McNaught & Mink, 2011; Patel et al., 2014). This is unfortunate, because, for a theoretical account, the phenomenology of TS is likely to be highly informative, as has been illustrated with respect to other psychopathologies, including social anxiety disorder (Gerrans & Murray, 2020) and major depressive disorder (Ramstead et al., 2023b). Individuals with TS report a rich set of sensory and cognitive symptoms, including canonical characteristics, such as tics, which possess their own unique (and complex) phenomenological variability (Jankovic, 1997). Indeed, tics are a multifaceted and unique set of behaviours that can vary from simple, reflex-like movements (e.g., eye blinking) to complex, compound, and seemingly goal-directed sequences of action that include several muscle groups (e.g., touching, sniffing, or jumping over an object). More generally, they have been described as “repetitive, patterned, and misplaced in context and time” (Beste & Münchau, 2018, p. 238).

Many of the sensory and cognitive symptoms of TS are shaped by ticcing behaviour and its expression, as well as its management through self-initiated coping strategies. Tics are often reported to be *“unwilled”* and not entirely voluntary (Cavanna & Rickards, 2013; Rae et al., 2019a). In a large portion of cases, the generation of tics is preceded by sensory phenomena called premonitory sensations (i.e., urges that precede tics), which are notably *“uncomfortable”* and require relief through action, i.e., via tics (Leckman et al., 1993). Furthermore, the deployment of inhibitory control over tics is said to be an *“effortful”* process (Brandt et al., 2016). However, there is considerable disagreement with respect to the interpretation of these phenomena within current studies of TS. For example, the volitional nature of tics remains a topic of contentious debate within the field (Cavanna & Rickards, 2013; Ganos et al., 2018; Haggard et al., 2002; Jankovic, 1997; Kwak et al., 2003; Lang, 1991; Rae et al., 2020). Without an overarching theoretical framework that accounts for both the experiential and behavioural characteristics of TS symptomatology, empirical results alone are unlikely to yield any clear resolution to such debates. Given that active inference often is proposed to be the grand unifying theory of cognition, it is at least a plausible framework through which the phenomenology and mechanisms of TS can be explained (Clark, 2013, 2015). We thus leverage active inference to offer both a synchronic analysis of the belief architecture of the ticcing individual and a diachronic account of that individual’s phenomenal and physiological trajectory.

In Section 1, we characterise tics as merely reflexive, active inference deployed as a perceived means of maintaining homeostasis. We describe a simple form of ticcing in response to premonitory sensation, whereby the release of a tic is likened to scratching an itch. In both scenarios, as a result of hypersensitivity to sensory data, overly precise prediction errors ascend the generative hierarchy, prompting the organism into action to suppress those errors, which are experienced as certain qualitative feelings (Barrett, 2017). This is consistent with clinical observations that TS patients have a “thinner barrier to stimulation” (Leckman & Cohen, 1999, p. 149). Crucially, we propose that tic behaviour effectively suppresses premonitory sensations, because it necessarily involves *sensory attenuation*. The effect is likely twofold: the organism actively changes their proprioceptive and interoceptive sensorium by ticcing, thus mechanically altering the likelihood distribution associated with the sensory data and minimising the prediction error at hand (i.e., the premonitory sensation); at the same time, the very act of ticcing—and action more broadly—engenders the attenuation of sensory afferents within the proprioceptive arc of the action, including, in the case of TS, those underlying premonitory sensations. This type of sensory attenuation through neuromodulatory (cf., precision weighting) control is necessary for motor behaviour in the first place.

In Section 2, we describe some ticcing behaviour as a form of epistemic foraging. We characterise this investigative form of tics (notably in the absence of premonitory sensations) as actions which serve to provide evidence of the existence of an agent. Specifically, *epistemic ticcing* can be construed as an attempt to resolve uncertainty about whether the “I” is the agentive cause of its own sensations, a belief that has been called into question because of the hyperprecision weight afforded sensory prediction errors, as described in Section 1. This follows because the failure to attenuate sensory precision weight entails the relative weakening of previous beliefs, which include the inferred capacities and intentions of the individual herself. We will suggest that this epistemic affordance is underscored by a subpersonal pragmatic preference to provide evidence for the reliability of one’s actions in causing certain outcomes, such that the epistemic tic does not only resolve ambiguity, but it also reinforces the belief that there is an agent at the heart of its generative (a.k.a., world) model. In summary, a propensity to tic within certain contexts is geared towards the discovery of evidence for the individual’s self-model, integrated therein by disambiguating the outcomes of self-generated action and worldly events (Clarke, 1996; Perrykkad et al., 2023).

In Section 3, we characterise both epistemic (sensation-free) and nonepistemic (sensational) tics as habitualised behaviour, cast as strong (habitual) prior beliefs about policy selection. This perspective sheds light on the subjectively effortless, or “*unvoluntary,*” nature of ticcing, as well as how tic behaviour might be influenced by negative emotions (Deane et al., 2024; Hesp et al., 2021; Parr et al., 2023). This is particularly salient within the context of adaptive behavioural control in TS, whereby the deployment of (intentional) inhibition to suppress ticcing behaviour is reported to be effortful, requiring intense attentional focus (Brandt et al., 2016; Rae et al., 2019a).

Finally, in Section 4, we propose that at the broadest, diachronic level of analysis, the aberrant action-perception loops—characteristic of TS—generate conflicting evidence for the state of the organism’s agency and self-coherence, leading to what we term *oppositional phenomenological self-modelling*. We propose that, in TS, the organism is drawn both *towards* ticcing as a way to minimise interoceptive prediction error, as well as *away* from ticcing because the action provides evidence against the higher-order preference that there is an socially sensitive, non-ticcing agent guiding the course of that organism’s life. This could be said to lead to a type of *duality of desires*.

In summary, we aim to show that this fourfold (active inference) account of TS symptomatology explains the diversity of TS phenomena, from the seemingly simple to the complex.

## Tourette syndrome

Tourette syndrome (TS) is a highly heterogeneous neurodevelopmental condition characterised by chronic and persistent tics (Rae & Critchley, 2022; Robertson et al., 2017). TS is assumed to be continuous with tics, a multifaceted set of behaviours that can vary from simple, reflex-like movements (e.g., eye blinking) to complex, compound and seemingly goal-directed sequences of action that include several muscle groups (e.g., touching, sniffing, or jumping over an object). In clinical contexts, tics are classified across dimensions, including time, type, frequency, location, and severity (DSM-IV; Leckman et al., 1989). Tics also carry a distinctive phenomenology in TS. Indeed, despite their intentional appearance, tics are experienced as “unvoluntary” and inflexible actions (Rae et al., 2020). Furthermore, tic expression is thought to be driven by many factors, including environmentally salient information (e.g., socioemotional triggers), suggestibility, psychological arousal, and autonomic status (Rae & Critchley, 2022; Rae et al., 2018). Hence, among hyperkinetic spectrum disorders, tics in TS possess the greatest clinical variability, reflecting a rich and complex range of human motor behaviour and its expression. Importantly, sensory phenomena also can be a prevalent feature of TS symptomatology. For example, tics are often preceded by uncomfortable urges or compulsions (i.e., premonitory sensations), which are, in turn, transiently relieved by the execution of the tic. Interestingly, the corresponding sense of relief resulting from tic release often is said to be akin to “scratching an itch” (Scholl et al., 2022; Singer, 2016). Finally, in many cases individuals with TS engage in self-initiated coping strategies and the management of tics (Matsuda et al., 2016). These include so-called tic suppression, an ability that allows those with TS to inhibit their tics through effortful, endogenous inhibitory control. Indeed, voluntary (i.e., intentional) inhibition appears to be intact in TS, whilst there is evidence of an asymmetry in automatic (i.e., reactive) inhibition in TS (Filevich et al., 2012; Morand-Beaulieu et al., 2017; Rawji et al., 2020).

Although it is not yet clear how to delineate precisely the complex symptomatology of TS in terms of its underlying (neuro)physiological mechanisms, neuroimaging studies have identified a degree of disconnection between frontoparietal networks (Naro et al., 2020).^1^ Much of the recent progress made on understanding the physiological basis of TS symptomatology has been facilitated by concurrent theorising about the complex phenomenological profile individuals with TS present. This literature has further benefited from the contribution of psychophysiological and other empirical, task-based approaches to TS, alongside computational approaches to modelling its symptomatology (Palminteri & Pessiglione, 2013; Rae et al., 2019a; Scholl et al., 2022). However, contemporary approaches maintain a primary focus on modelling the overt symptoms of TS symptomatology (e.g., tics and their formation as habitual behaviour) whilst overlooking many of the covert, qualitative characteristics of TS and its expression. These include premonitory sensations and the autonomic, contextual influences of stressors within the social and cultural environment on the expression of tics and (dis)inhibition. For example, emotional variables are known to influence ticcing behaviour, whereby negatively valenced states of anxiety or stress are perceived by those with TS to intensify their ticcing (Bornstein et al., 1990; Eapen et al., 2004; Leisman & Sheldon, 2022; Silva et al., 1995). In many cases, this co-occurs with even stronger and more uncomfortable premonitory sensations.

### Tic-prone brain states in TS

Whilst the occurrence of tics is transient and bound to particular events, empirical studies have suggested the presence of “tic-prone brain states” in TS, which predispose the individual to certain habitual behaviours and facilitate tic expression (Rae & Critchley, 2022). More specifically, a well-documented impairment of the interactions between limbic, paralimbic, and cortico-striatal-thalamo-cortical pathways (i.e., CSTC loops) has been shown to underwrite the pathophysiology of TS (Naro et al., 2020). In particular, neuroimaging evidence highlights the role of interactions between the basal ganglia, presupplementary motor area (preSMA), and affective, motivational regions of the insula in generating ticcing behaviour and its expression (Brass & Haggard, 2010; Jackson et al., 2015; Rae et al., 2018).

Indeed, the basal ganglia, which is constituted, in part, by the striatum, interfaces with cortical regions via the thalamus, forming CSTC loops that effectively gate the context-sensitive initiation of movement. Regulating the relative balance of neuronal excitation-inhibition during voluntary action and its control involves the so-called *direct*, *indirect*, and *hyperdirect* pathways of the basal ganglia. Within the direct, excitatory pathway, the globus pallidus (internal) and the substantia nigra pars reticulata (SNpr) are inhibited by striatal medium spiny GABAergic neurons, allowing for disinhibition of the ventral anterior (VA) and ventrolateral (VL) nuclei of the thalamus and the *promotion* of movement (i.e., excitatory signals propagated from thalamus to cortex). Within the indirect, inhibitory pathway, the globus pallidus (external) is inhibited by the striatum, allowing for the disinhibition of the glutamatergic neurons of STN. This activates SNpr and global pallidus (internal) GABAergic neurons, which project to the VL and VA nuclei of the thalamus and, ultimately, *prohibit* movement. Finally, a hyperdirect pathway enables the *pausing* of movement, with direct connections between the cortex and STN that feedback to globus pallidus (internal) and prompt inhibitory signals to thalamus (Calabresi et al., 2014; DeLong, 1990; Friend & Kravitz, 2014; Nambu, 2004; Rae & Critchley, 2022; Young et al., 2024).

Dysfunction of these pathways has been shown to underwrite a number of movement-related pathologies, including Parkinson’s disease (Blandini et al., 2000) and Huntington’s disease (Starr et al., 2008). With respect to tic behaviour, glutamatergic inputs from the supplementary motor area (SMA) to the striatum alongside a reduction in striatal inhibitory interneurons have been proposed to amplify activity within the direct pathway of the basal ganglia, which leads to thalamic disinhibition and the release of movement signals to M1 (Albin & Mink, 2006, Conceição et al., 2017; Ganos et al., 2013; Neuner et al., 2013; Mink, 2001; Polyanska et al., 2017; Rae & Critchley, 2022; Rae et al., 2018; Redgrave et al., 2010; Singer, 2005; Zapparoli et al., 2015). However, in TS, there is evidence of atypical interactions across many other areas, including premotor cortex, insular cortex, and S1 (Bohlhalter et al., 2006; Neuner et al., 2014; Polyanska et al., 2017; Wang et al., 2011). For example, the insular cortex (a still largely enigmatic system, see Uddin et al., 2017) has been proposed to play a common yet overlooked role in TS, especially as a potential substrate for premonitory sensations—the characteristic “feeling-states” associated with sensory discomfort and compulsions—which commonly precede the generation and expression of tics (Bohlhalter et al., 2006; Cavanna et al., 2017; Conceição et al., 2017; Neuner et al., 2014; Rae et al., 2018). Indeed, the grey-matter thickness of the insula has been shown to be negatively correlated with the intensity of premonitory sensations (Draper et al., 2016), as measured by the Premonitory Urges for Tics Scale (PUTS) (Reese et al., 2014). Furthermore, the strength of functional connectivity between the anterior insula and the SMA (at resting state) has been shown to positively correlate with high PUTS scores (Tinaz et al., 2015). In Section 1, we go beyond the broad-brush account provided here and offer a more fine-grained analysis of the dysfunctional interactions within CSTC loops—with a particular focus on the connection between the insula and the SMA—which underscore sensation-based ticcing behaviour.

Finally, some individuals with TS are able to engage in “tic suppression,” which refers to the highly “effortful” deployment of inhibitory control to withhold tics. This ability has been documented within neuroimaging literature in an attempt to better understand the neurocognitive processes that influence ticcing behaviour. Here, tic suppression is associated with effortful, top-down modulation, specifically through inhibitory control processes that exploit a hyperdirect pathway, primarily implicating the inferior frontal gyrus, preSMA, and subcortical networks involved in action control, such as the basal ganglia (Aron et al., 2016; Ganos et al., 2014; Rae et al., 2015, 2019a, 2020).

Given the structure of interactions across CSTC loops and their integrated nature, it has been shown that hierarchical mechanisms can be applied to successfully model the known pathophysiology of ticcing behaviour in TS and its links to phenomenology (Rae et al., 2019a). We thus examine the consistency of this hierarchical model within the overarching theoretical framework of active inference to account formally for the sensory, cognitive, and motor symptoms of TS. It is to this framework that we now turn.

## Active inference

For those familiar with the active inference framework (AIF), the following section can be passed over. However, given that we utilise the framework in our explanation of TS, it is worth unpicking its background theory somewhat broadly, before outlining how it can be used as an explanatory tool in the analysis of TS. Note, we have chosen not to cover every detail of active inference for simplicity’s sake (cf., Friston, 2019; Ramstead et al., 2023a for a comprehensive treatment of the framework).

Active inference is a corollary of the Free Energy Principle, which holds that any *thing* that persists through time—in so far as it frequents a limited set of characteristic states—attains a nonequilibrium steady state (Friston, 2009, 2010, 2019; Friston et al., 2017a; Kirchhoff et al., 2018; Ramstead et al., 2023a). In doing so, such things look as if they are performing (variational) Bayesian inference—insofar as internal states (e.g., neuronal activity) can be read as modelling external states (e.g., the physiological state of the body or states of affairs in the environment). More specifically, the ensuing behaviour can be read as maximising evidence for a generative model that the existence of the entity entails, which also can be cast as minimising variational free energy, an information theoretic term that acts as an upper bound on surprisal or Shannon self-information. In the active inference literature, this process often is called “self-evidencing” (Hohwy, 2016).

Note that, at least in energetically bound entities like humans, this process cannot involve true Bayesian inference, because two quantities—the model evidence *P*(*o|m*) and the posterior *P*(*s,u*|*o*)—cannot be computed because of the indefinite number of hidden states that need to be marginalised and because such marginalisation requires integrals that are computationally intractable. As is common within the AIF, *o* stands for observations, *u* are actions, and *s* are latent states. Thus, we can only conduct approximate Bayesian inference, whereby the true posterior is replaced by a tractable (Bayesian) belief known as recognition density (*Q*) and the accuracy of that approximation can be expressed in terms of a Kullback-Liebler (KL) divergence. Mathematically, minimising variational free energy entails both minimising this KL divergence—which can be read as *perception*—and then *acting* to sample new observations to ensure the maximisation of model evidence: *P*(*o|m*). This permits the formulation of the equation for variational free energy *F*, as a functional (function of a function) of the recognition density *Q* and observations *o* (Equation 1).

Equation 1. Variational free energy equation.

$$\begin{array}{lc}Q\left(s,u\right)=\text{argmin}_{Q}\;F\\\ \ \ \ \ \ \ \ \ \ F=E_Q[\text{In}\underbrace{\;Q\left(s,u\right)}_{posterior}-\text{ln}\underbrace{\;P\left(o\left|s,u\right.\right)}_{likelihood}-\text{ln}\underbrace{\; {P(s,u)}}_{prior}]\\\ \ \ \ \ \ \ \ \ \ \ \ \ \ =\underbrace{D_{KL}\left[Q(s,u)\parallel P\left(s,u\left|o\right.\right)\right]}_{divergence}-\underbrace{\text{ln}\;P\left(o\left|m\right.\right)}_{log\;evidence}\\\ \ \ \ \ \ \ \ \ \ \ \ \ \ =\underbrace{D_{KL}\left[Q(s,u)\parallel{P(s,u)}\right]}_{complexity}-\underbrace{E_Q\left[\text{ln}\;P\left(o\left|s,u\right.\right)\right]}_{accuracy}\end{array}$$

In complex creatures, such as humans—who infer states of the world given sensory impressions upon exteroceptive, interoceptive, and proprioceptive modalities—it has been proposed that the minimisation of variational free energy for the organism at large occurs via a (temporally) deep, (hierarchical) generative model, in which “higher,” more abstract levels track slower, regular flows in the external states and contextualise and constrain “lower,” more context-bound levels, which are (statistically) attuned to rapid environmental dynamics. This has frequently been described from a predictive coding, or more broadly, a predictive processing perspective, whereby prediction errors at a lower level are dynamically shuttled up the cognitive hierarchy and are “explained away” by higher levels, which are issuing cascading predictions (Clark, 2013, 2015).^2^

Technically speaking, a prediction error is the divergence between my prior belief—the probability of being in a state *s*—and my posterior belief—the probability of being in a state *s,* given an observation *o*. Upon registering a prediction error, I can resolve it by either updating my beliefs, so that my posterior provides an apt explanation of the observations, or change the way I sample the world through action, so that my observations aligns more closely with my prior expectations. However, the very presence of this binary decision is predicated on the fact that a prediction error has sufficient (precision) weight (cf., postsynaptic gain) to force me to (subpersonally) make a choice; otherwise, I can simply ignore it. Thus, an important feature of the implicit cognitive, predictive dynamics is so-called precision weight. Precision is technically the inverse dispersion of a probability distribution and can be understood as the confidence afforded to probabilistic (Bayesian) beliefs. For example, perception entails the inversion of the generative model such that the predictive system infers a specific hidden state (s) given a sensory observation (o) (cf., Kalman filter). If the likelihood in Equation 1 has high precision—or low entropy—then the system will have high confidence in its inference: i.e., that *o* was truly caused by *s*. The likelihood is generally parameterised within the A tensor in POMDP schema. An observation with high precision implies a precise, unambiguous mapping between cause and consequence, whereas for a low precision observation, a multiplicity of causes might underlie it. Crucially, there is a difference between sensory precision, as a purely mathematical construct connoting the inverse dispersion of a probability distribution, and sensory precision weight, which refers to how much postsynaptic gain a signal is afforded and, thus, how much it biases the posterior in its direction, given that the posterior is a sort of compromise between the likelihood of a sensory signal (given a hypothesised state) and the prior expectation of that state. Precision certainly factors into precision weight, because minimising variational free energy entails accurate inference of the true hidden state that caused an observation, it is not the only factor at play (cf., Parvizi-Wayne, 2024 for a thorough discussion of this topic). It is for this reason that we will refer to precision weight rather than precision when discussing the postsynaptic gain of signals per se.

In the setting of continuous state space, which is typically used to model low-level sensorimotor activity, a precisely weighted likelihood corresponds to a precisely weighted prediction error, which has a greater effect on belief updating higher in the hierarchy. Physiologically, this is thought to rest on neuromodulatory mechanisms that tune postsynaptic gain of neuronal populations reporting prediction errors. The complement of postsynaptic gain is sensory attenuation: namely, a reduction in the precision weight of sensory prediction errors (or, more generally, sensory likelihoods). Sensory attenuation is an important faculty in the context of active sensing and motor control in the following sense: it enables individuals to ignore sensory evidence that they are not moving and thereby realise prior beliefs that correspond to the anticipated sensory consequences of intended movements (Adams et al., 2013; Brown et al., 2013). Perhaps the best example of this is saccadic suppression: namely, the loss of precisely weighted visual prediction errors during saccadic eye movements. This precludes our perception of the ensuing optic flow and permits swift and graceful palpation of the visual scene. In short, any volitional movement is accompanied by sensory attenuation—i.e., an attenuation of sensory precision weight.

It is important to note that precision weight can be granted to beliefs encoded in a generative model, including beliefs about how the world evolves (parameterised by the B tensor in POMDP schema), prior expectations over sensory outcomes (encoded by the C tensor), and prior beliefs about policy selection (E tensor). Recent work has suggested that inferring and deploying these second-order beliefs (i.e., beliefs about the precision weight of beliefs) underwrites mental action (Limanowski & Friston, 2018; Sandved-Smith et al., 2021). An explication of the (aberrant) dynamics unfolding among these second-order beliefs is required for the active inference account of TS that follows.

## Fourfold analysis of active inference and TS symptomatology

### Section 1: A (believed) restoration of homeostasis

**Fig. 1:**
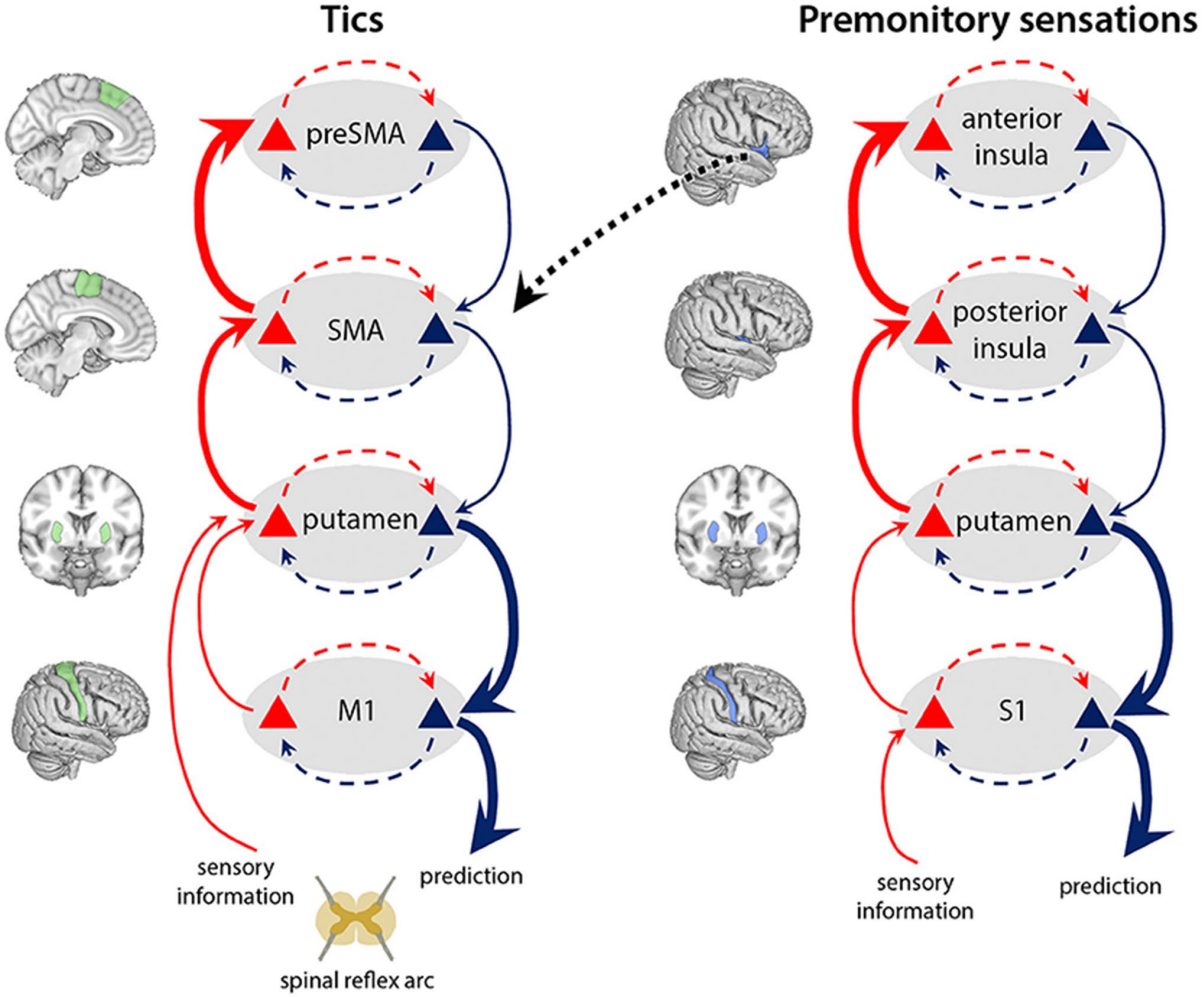
The model of TS used by Rae et al. (2019a), within which tics and premonitory sensations are generated via aberrant precision weighted prediction dynamics in a predictive hierarchy. Reprinted from Rae et al. (2019a)

With this background in place, we can describe a simple form of ticcing, namely those preceded by premonitory sensations, as enacting a form of (technically) Bayes-optimal action selection, albeit with precision weighting dynamics that lead to broadly maladaptive consequences. To this end, we will draw upon the preexisting model of TS from a Bayesian perspective constructed by Rae et al. (2019a). These researchers ground the generation of tics and premonitory sensations in two respective modulations of the precision weighting of the generative model. These are abnormally precisely weighted priors for action—technically the habit, or E tensor, in POMDP schema—and abnormally high sensory precision weight (Fig. 1). Note that, with respect to the latter, Rae et al. (2019a) do not specify whether this constitutes aberrant precision weight over preferred sensory observations—C tensor—or the likelihood distribution—A tensor. In the variant of the model that we employ, we will focus on the likelihood precision weight, in relation to the precision weight of prior beliefs about state transitions (B tensor), although we recognise that prior preferences (C tensor) and initial state priors (D tensor) will invariably have a role to play. We will leave our discussion of habit formation in TS to Section 3.

It is important to note that not all tics are associated with premonitory sensations; some estimates suggest up to 40% of tics occur in their absence (Leckman et al., 1993). This fact motivates a theoretical dissociation of the sensory and prior precision weight, because one need not experience sensory symptoms, putatively generated through ascending prediction error from the putamen to the anterior insula, to engage in ticcing behaviour. Indeed, according to Rae et al. (2019a), tic frequency is (merely) ramped up by the signals sent from the anterior insula to the SMA; however, given that premonitory sensations are not necessary for tic behaviour, they propose that, in TS, there is already overactivity in the SMA and reduced GABAergic interneurons in the putamen, leading to the disinhibition of the thalamus, the activation of M1 and the production of tics, as described in §2.1. However, in this section, we will focus on those tics that are driven by uncomfortable sensations.

According to Rae et al. (2019a, 2019b), TS involves hyperprecise sensations, generated at the posterior insula, which are “explained away” at the anterior insula as unwanted feelings (i.e., premonitory sensations). For example, in a case reported by Bullen and Hemsley (1983), a patient with TS (“J”) described a “horrible” sensation in her upper back that she could only “get rid of by moving” whilst undergoing habit reversal therapy. The closest analogy to this sensation was to compare it to an “itch.” We read this as the consequence of hyperprecision weighting over the A (likelihood) tensor, which statistically draws the posterior away from the prior density. This increased precision weight over the likelihood of the sensation maps onto an increase in prediction error—cast as the divergence between prior and posterior densities. To reduce this prediction error, it is incumbent on the model the organism embodies to achieve *both* perceptual adequacy—technically the minimisation of the KL divergence between the true posterior and the recognition density *Q*—and normative adequacy—the minimisation of surprise. Whereas the latter is realised (both phenomenologically and computationally) by the state inference that there is an uncomfortable feeling at hand, the latter occurs through motoric action, engendered by a prediction to not be in this uncomfortable state. To resolve the present prediction error, therefore, the anterior insula sends signals for action to the SMA, leading to tic generation via descending proprioceptive predictions (i.e., glutaminergic inputs) to the motor cortex and putamen (Somogyi et al., 1981). In POMDP schema, this might be cast as the result of high precision weight over the C tensor, which encodes the preferred outcomes of action. To return to patient J, it was reported that the itch (i.e., prediction error) disappeared (i.e., the resolution of prediction error) once such movement was initiated, giving the individual a temporary sense of relief.

Crucially, we propose that this resolution of prediction error through sensory attenuation is likely twofold. To a certain degree, the prediction error might be reduced directly and mechanically by the mere act of ticcing, which changes the proprioceptive and interoceptive state of the organism such that the sensory data it receives now accords more strongly with its prior expectations. From this perspective, ticcing behaviour can be seen as the result of a failure of sensory attenuation in a manner different from how such a failure putatively underwrites movement disorders (Brown et al., 2013; Pareés et al., 2014; Seth, 2014), and is strongly akin to scratching an itch.

Conversely, the type of sensory attenuation that, according to active inference, *necessarily* accompanies the emission of any action is also implicated in ticcing. This is because action involves the attenuation of sensory afferents, which otherwise indicate to the organism that they are not moving, as explained above, whilst higher precision weight is granted to the *final* sensory consequences of an intended action (Adams et al., 2013; Brown et al., 2013; Limanowski, 2017; Parr et al., 2018). Crucially, in the case of tics, these target afferents include those underlying premonitory sensations, because they belong to an anatomical region that constitutes part of the (predicted) action arc of tics. In other words, there is strong overlap between where one tics and where one feels premonitory sensations. Given that, to be able to act at all, the precision weight over the sensory data - whether proprioceptive, interoceptive or exteroceptive - belonging to the (current) relevant action arc needs to be attenuated, ticcing, *by necessity*, entails reduced precision weight over the interoceptive prediction errors constituting premonitory sensations, thereby alleviating the individual’s discomfort.

In summary, at this sensorimotor level, we hold that, due to synaptic dysfunction, premonitory sensations are the result of an abnormally high level of precision weighting deployed onto the A tensor (i.e., weighted precision error), which draws the organism to act to minimise the variational free energy of their generative model. Note that given the relationship between the A tensor and attention, it is no surprise that premonitory sensations are said to powerfully occupy the mental lives of those suffering from TS (Martino et al., 2013). To resolve the prediction error yielded from hyperprecision weight over the A tensor, the organism must act to engender the necessary sensory attenuation. We hold this active response likely involves two processes: the mechanical, direct alteration of sensory data; and the modulation of precision weight entailed by action itself. The degree to which one type of sensory attenuation predominates over another in ticcing is an open question and ought to be answered empirically.

### Section 2: Tics as epistemic foraging

In the BBC series, “The Mind Traveller” with neurologist Oliver Sacks (Rawlence et al., 1998), we are introduced to Shane Fistell, who has TS, and characterises his tics as a way of disambiguating the limits of, for example, social norms, and describes ticcing behaviour as “at its highest… a form of investigation… and it could be also an emotional, not just physical, investigation.” This seemingly introduces a different motivation underlying tics: namely, information gain rather than the maximisation of pragmatic value through sensory attenuation. We propose that in many of the aforementioned contexts when tics occur in the absence of premonitory sensations (Leckman et al., 1993), it is this type of *epistemic* tic at play.

More precisely, we hold that the propensity to tic within certain contexts serves to resolve ambiguity about the status of the inferred, agentive self by disambiguating the outcomes of self-generated action and worldly events (Clarke, 1996). This crucially involves both information gain about the agent “that I am” and the fulfilment of a supervening pragmatic imperative, which draws me to act so that “I will be able to observe the sensations that I cause”—perhaps the most overt expression of self-evidencing (see e.g., self-stimming behaviour in severe autism (Nwaordu & Charlton, 2023).

This need to buttress my belief—about the type of agent that I am—stems from the failure of sensory attenuation outlined above. This is because unduly highly weighted sensory signals will necessarily attenuate the relative precision weight of state priors (encoded in the B tensor), which, in turn, will act as evidence for the higher-order inference that the latent states that I encounter (my world) are unpredictable, imprecise, and volatile. Crucially, this belief in volatility also is self-referential, because, in active inference, the self is cast as just another latent state or cause of multimodal sensory information—albeit a unique one in its capacity to stably bring about the precise sensory expectations the agent has of her actions (Deane, 2020; Deane et al., 2020; Deane, 2021; Hohwy & Michael, 2017; Friston, 2018; Limanowski & Blankenburg, 2013; Limanowski & Friston, 2020). In other words, the self has been described as a constructed hypothesis, resulting from an inference that there is a stable, persisting *thing* at the heart of the cognitive system, which can autonomously bring about desired (predicted) sensory outcomes (Metzinger, 2004, 2008, 2013).

The maintenance of this stable sense of selfhood therefore is contingent on the alignment of predicted and observed sensory data, which means that the observations resulting from self-generated action need to be minimally surprising, given the individual’s generative model (see Equation 1) (Frith, 2012; Hohwy, 2007). With weak priors and strong likelihoods in play, however, the precision weight of the posterior belief about the cause of sensation—in this case, that it was the agentic being I call me—is reduced with accompanying uncertainty. This engenders a pernicious cycle in which the continuous aggregation of uncertainty continues to reduce the precision weight of the individual’s belief about their agentive nature. Although confidence in that belief will be, in part, reinforced by the individual’s ticcing, which, recall, is aimed at sensory attenuation, we propose that many individuals suffering from TS will take further steps to seek evidence of their own agency and autonomy.

Thus, we introduce the notion of *epistemic ticcing*, which we suggest is motoric action designed simultaneously to test the individual’s capacity to bring out about a predicted sensory outcome—thereby yielding information gain about the hidden causes of which “I” am one—and to reinforce the belief that, indeed, there is an agent within the generative model that can bring about the sensory outcomes it predicts will result from its action. This means that there is a utility achieved through epistemic ticcing, which underwrites its associated information gain. In other words, the individual only seeks to gain information about the status of their control to provide evidence for an overarching preference that they actually are creatures with agency. Our proposal here is consonant with the findings of Perrykkad et al. (2023, p. 617), who show experimentally that “participants can learn to use environments to their advantage in facilitating unequivocal judgements of agency,” given that uncertainty in the belief of one’s own agency impedes the pursuit of other rewards, whether they be instrumental or epistemic (Inglis, 2000).

### Section 3: Habitualised tics and their connection to valence

It is noteworthy that, as mentioned earlier, certain individuals can suppress their tics as a self-initiated coping strategy (Ganos et al., 2014; Matsuda et al., 2016; Rae et al., 2020; Rawji et al., 2020). However, such inhibitory control is said to be effortful and tiring (Brandt et al., 2016). In fact, for some individuals the sensory discomfort and/or effort of suppression is so unpleasant that it symptomatically outweighs the tic itself (Stern, 2018). For example, one young person with TS summarises the subjective experience of suppressing tics as follows:

“…I’m holding them in, it puts pressure on me…that makes me stressed and then makes me upset…” (Smith et al., 2016).

This is of particular relevance from an active inference perspective, because it has recently been proposed that a sense of cognitive effort is rooted in the selection of a policy^3^ that diverges from prior beliefs over policies—i.e., what I would normally do in this situation (Parr et al., 2023). Priors over policies are thus associated with habits in active inference, which are acquired by the individual performing an action policy and subsequently inferring what action was taken in a given context. This information can then be stored as a prior probability, or value, over policies associated with specific states (encoded in the E tensor in POMDP schema) such that when the individual infers itself to be in a given context, the prior value of an action policy influences the selection of the policy itself (Maisto et al., 2019). This is because the total probability of a policy is determined by a combination of priors before (E) and after evaluating EFE (G) (Equation 2).

Equation 2. Contribution of Prior Values and EFE to Action Selection.

$$\begin{array}{c}Q\left(u\right)=\sigma \left(E-G\right)\end{array}$$

*Note that the σ notation refers to a normalised exponential - i.e., SoftMax - function.*

This equation shows that the most likely action—i.e., the one that possesses the highest posterior probability *Q*(*u*)—has a high prior habitual value *E* and a low associated EFE *G*, which recall, is in part predicated on preferred sensory observations (encoded by a C tensor or its parameters *c*).

The role prior, habitual values have in action selection is closely tied to the notion of “canalisation,” as described by Carhart-Harris et al. (2023), who repurpose the original meaning of the word—phenotypic stabilization (Waddington, 1942, 1959)—so that it now refers to the entrenchment of cognitive and behavioural patterns over time through associative (Hebbian) learning that strengthens neural connections (Cicchetti & Tucker, 1994; Deane et al., 2024). As Deane et al. (2024, p. 6) nicely put it: “many disorders feature the pathological stabilization of particular cognitive–behavioral pathways, reflecting their shared reliance on (Hebbian) neuroplastic processes of canalization gone awry.”

Like Deane et al. (2024), we propose that habits may underscore pathological behavioural processes, which we explore within the context of tics. Our central point is that the more the individual responds to premonitory sensations through tic behaviour, the greater the habitual prior of that tic policy becomes in association with that state of inferred discomfort. It is for this mechanistic reason that ticcing behaviour seems so effortless as to be *unvoluntary* (Rae et al., 2019a). This description is useful, because it captures the strength of the canalised ticcing behaviour; however, it does not preclude the possibility for top-down control that certain individuals can evidently deploy to suppress tic behaviour. That said, it has been noted that such top-down inhibition is incredibly effortful (Brandt et al., 2016; Rae et al., 2019a, 2023). We show that such inhibitory control within TS can be accounted for through the AIF by recognising that tic suppression involves action selection that is divergent from what the individual would normally do in that context—namely, tic. In other words, tic suppression possesses a low habitual prior in that inferred state, unlike tic behaviour. When the tic is not suppressed, the posterior over action is dominated by habitual priors. This means that the divergence between the posterior and prior over action is small and—on the formal arguments that this divergence corresponds to effort— the path of least effort is to select the tic behaviour.

Finally, it is worth mentioning that canalised behaviour arguably underwrites both tics aimed at sensory attenuation and epistemic foraging. Whilst the contextual cue for the former is often the presence of the premonitory sensation, the trigger for the second type of behaviour might be an inferred loss of precision weight over beliefs about the capacity for autonomous control and agency.

We can embellish this story further by folding in the notion of action precision weight, γ, a parameter in the generative model that tracks beliefs the agent has in its own action selection (Deane et al., 2024; Hesp et al., 2021). More precisely, this gamma parameter is said to scale EFE estimates, formalised by Equation 3, which is a modification of Equation 2.

Equation 3. How action precision weight influences the contribution of EFE to policy selection.

$$\begin{array}{c}Q\left(u\right)=\sigma \left(E-\gamma\cdot G\right)\end{array}$$

*Note that the “dot” notation indicates a backwards matrix multiplication and normalisation.*

Importantly, Hesp et al. (2021) propose that γ can be treated as a hidden state within the generative model, such that the system can infer its own confidence estimate in its action model, and that this state inference corresponds to positive and negative valence. Put another way, if an individual infers herself to have high confidence in its action selection, it will *feel* good (i.e., confident in the sense “I know exactly what to do next”); if it does not, it will feel bad (i.e., the angst that attends “I don’t know what to do in this situation”) As Equation 3 shows, as gamma tends to zero—as low affect grows—action selection becomes dominated by *E* because (negative) EFE is multiplied by gamma in its contribution to *Q*(*u*). This might explain why negative emotions, such as anxiety, stress, tension, and frustration, are associated with tics (Bornstein et al., 1990; Eapen et al., 2004; Leisman & Sheldon, 2022; Silva et al., 1995). Given that negative affect biases policy selection towards habits (*E*) and that tic behaviour can be cast as fundamentally habitual, it is expected that as negative affect builds, ticcing becomes even harder to resist. Note that, perhaps for reasons we will suggest below, a lower quality of life is strongly associated with TS (Eapen et al., 2016). This means that a pernicious cycle is likely at play in TS, whereby habitualised tic behaviour drives low affect, which drives tic behaviour and so on. Interestingly, changes in gamma have been associated with dopaminergic firings in the midbrain (Hesp et al., 2021; Friston et al., 2015; Schwartenbeck et al., 2015). This aligns with the aforementioned mediating role of dopamine with respect to tic expression in negatively valenced states of anxiety or stress, because such contexts will entail the “reporting” of negative prediction error—i.e., a drop in gamma—via dopaminergic pathways (Schultz, 2016; Schultz et al., 1997).

It is worth clarifying that, in terms of these low-level sensorimotor loops, the action policy that individuals would otherwise select on the basis of their preferred sensory observations would likely not be strongly divergent from the habitualised tic action. The tic only became habitualised (i.e., obtained a high prior value) because it was selected initially either as a result of its inherent pragmatic value in suppressing premonitory sensations (with respect to the tics described in Section 1)  or its combined epistemic and pragmatic value (with respect to the tics described in Section 2). Thus, if we only look at this level of the generative hierarchy, we can plausibly suggest that beliefs encoded both in the C tensor and the E tensor drive tic behaviour. If this is the case, it means that the reason why tics become more frequent in negatively affective states cannot be because of the reduced influence of EFE in action selection in and of itself. Rather, habitualised behaviours might become more impervious to top-down control: i.e., the beliefs encoded in high-level C tensors. It is to these higher-order priors that we now turn.

### Section 4: Self-modelling and TS

In this section, we shall adopt a broad, diachronic perspective and explore how tic behaviour can gradually erode the prior beliefs the individual entertains about herself, which are putatively encoded at the deepest (or highest) levels of the generative hierarchy and thus *should* act as constraints over the unfolding of lower-level sensorimotor behaviour (Clark, 2013, 2015; Hohwy & Michael, 2017). Before doing so, it is worth examining the hierarchical structure of a generative model that putatively underwrites the ability to reflect on oneself as a self – what Parvizi-Wayne et al. (2024) call a *conceptually-represented-self-as-object* (cf., Gallagher & Zahavi, 2020). 

As mentioned, the deeper levels of the hierarchy are said to track slower narratives, trajectories, or fluctuations in external states. Crucially, encoded in these neuronal pathways (i.e., the neuronal instantiation of the tensors in POMDP formulations) will not only be beliefs about worldly contingencies, such as seasonal changes, but also beliefs about “endogenous”, interacting, latent, long-term patterns (or causes), such as personality traits, likes and dislikes and so on, which map onto (or engender) patterned sensory data from which those latent, internal patterns/causes can be inferred (Hohwy & Michael, 2017, p. 8). This is because, in terms of the generative modelling utilised by the active inference framework, these beliefs are not considered fundamentally different from those that target states in the world, since, as mentioned in Section 2, both the self and the world are cast as *inferred* latent causes of sensory observations (cf., Deane, 2021; Hohwy & Michael, 2017; Limanowski & Friston, 2020; Woźniak, 2018). Thus, it has been proposed that the target of these self-referential beliefs are “deeply hidden internal causes of the agent” (Hohwy & Michael, 2017, p. 7), and that the (hierarchical) net of these beliefs constitute the represented self, or self-concept.^4^ In other words, when these higher-order beliefs are aggregated, they can be said to answer the question: “what type of agent am I?” (Hohwy & Michael, 2017, p. 9). Note that this conceptual self-as-object is often termed the narrative self; however, it is worth recognising that this narrational aspect to the generative hierarchy need not be entirely coherent or linguistic (Dennett, 1991; Strawson, 2004); rather, as put by Hohwy and Michael (2017, p. 9), they are narrative “in the sense that the subsumption of events under higher-level regularities (i.e., hierarchical Bayes) structures and constrains our interpretation of those events.” We simply add to this elegant summary that, if the individual is to continue being the thing that it is, those higher-level regularities should also constrain the actions deployed at lower sensorimotor levels, because higher levels issue predictions about lower-level beliefs, which are fulfilled through action.

Crucially, however, we need not remain exactly the same thing into the future (Clark, 2017). Rather, we have (often personal, linguistic) preferences about the types of things we want to be, which might be different from what we perceive ourselves to be now (Rosfort & Staghellini, 2009). Put differently, what might be termed the *veridical self-concept* might differ from the* desired self-concept*. Importantly, this type of temporally extended, dynamic self-modelling will be shaped by individuals’ cultural surroundings, including their community’s language (Nelson, 2003), social norms (Gilbert 1990; Searle 1995), and the general behaviour of proximate conspecifics (Gergely et al. 2002; Meltzoff, 1995). Given the perceived pathological status of TS (Hirschtritt et al., 2015), as well as the prejudice faced by those who suffer from it (Malli & Forrester-Jones, 2022), its (inferred) presence is unlikely to be a desired feature within a given individual’s generative model.^5^ This intuition is buttressed by the fact that those with TS suppress and conceal their tics to avoid being labelled disabled (Buckser, 2008) and characterise their syndrome with the negatively valenced notion of difference: i.e., as something that prevents them from being normal (Malli et al., 2019). To quote Participant 15 from the qualitative study of Malli et al. (2019):

*“It's a disorder that just makes me, separates me from the rest, and in my eyes, it's quite a big distance, because I don't feel as though I can live a normal life most of the time with it. And it's not even just things that people can see on me when they first meet me. They are the things that happen behind closed doors that no one sees and only I see, or sometimes don't see.”*

Note that the study of Malli et al. (2019) discusses other reasons why TS sufferers adopt a negative attitude towards the syndrome; however, for the sake of this theoretical analysis, it suffices to say that, in terms of explicit *desires*, supposedly encoded at the higher, more fixed levels of the generative hierarchy, individuals with TS would prefer not to exhibit (ego-dystonic) tic behaviour and execute tic suppression in line with this (ego-syntonic) preference. In active inference, preferences and beliefs are elided, whereby an agent’s preference for a course of action can be described as a belief about what it will do, or, more precisely, the future states it will find itself in (Friston et al., 2014; Friston et al., 2015). Thus, we can plausibly propose that TS sufferers possess a higher-order belief that they will not enact their tics in line with their expectations about what socially situated agents should be like.

However, given what we have said about tics deriving from sensorimotor preferences to mitigate failures of sensory attenuation and/or gain information about the state of the individual’s capacity for autonomous control—and that these beliefs continue to be held even after ticcing becomes canalised—it now becomes plausible to suggest that TS involves a clash of preferences, or a duality of desires. This is because at higher-levels of the generative hierarchy the individual prefers not to tic, in order to maximise evidence for the belief in (them being) a non-ticcing, socially acceptable agent. However, at lower-levels tics are sub-personally and implicitly preferred, because they possess pragmatic or epistemic value, given the belief architecture at that level of the generative model. Crucially, we propose that this maps onto the phenomenology of selfhood: whereas tic suppression will provide model evidence for a belief in the presence of a certain, desired (aspect of a) self-concept (i.e., I am not a ticcing thing), it prevents the realisation of the (perceived) need for sensory attenuation. This second point is pivotal because interoceptive inference, achieved through sensory attenuation, has been argued to underwrite a minimal sense of self or minimal phenomenal self (MPS) (Barrett & Simmons, 2015; Blanke & Metzinger, 2009; Critchley & Seth, 2012; Limanowski & Blankenburg, 2013; Metzinger, 2008; Seth, 2013; Seth et al., 2011; Suzuki et al., 2013). For example, Blanke & Metzinger (2009, p. 8) write:

*“The essence of selfhood, the representational content of MPS, is that it indicates to the system that a specific state in its internal neural dynamics has been reached, namely an integrated functional state, which for the first time makes the body available for attention and global control. MPS, characterized by self-identification, self-location, and weak 1PP [1st person perspective], is the outcome of this process on the level of conscious experience.”*

Similarly, Critchley & Seth (2012, p. 425) write:

*“Self-consciousness is grounded on the feeling states that emerge from the interaction of interoceptive predictions and prediction errors.”*

**Fig. 2:**
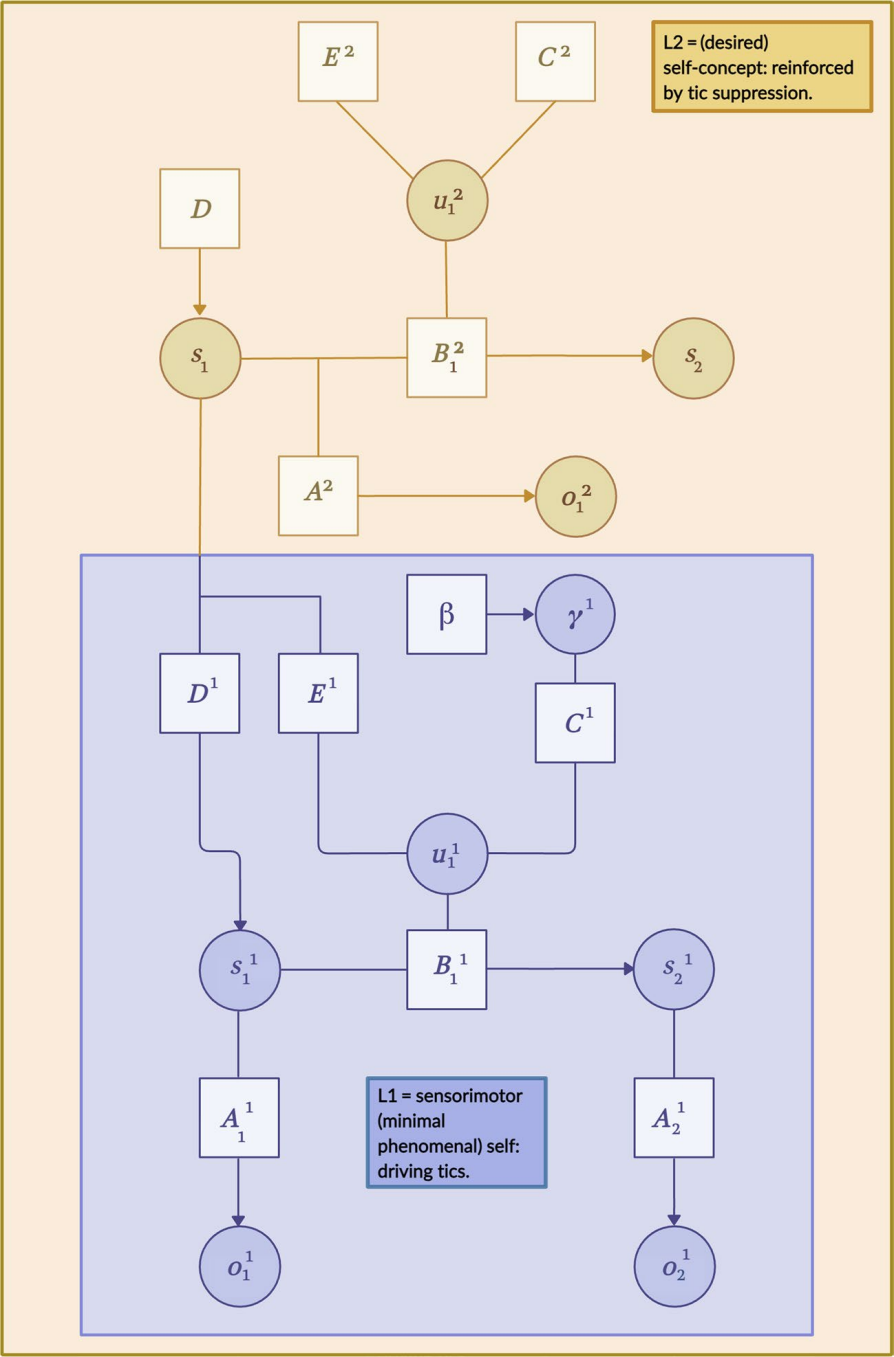
Hierarchical generative model of OPSM in TS. A hierarchical Bayes graph representing the inferential architecture that we propose is responsible for the experience of OPSM in TS. Some dependencies (i.e., edges) have been omitted for clarity. Arrows indicate the direction pf dependencies between variables. Shaded circles represent inferred beliefs about states s and actions u given observations o and the parameters of the generative model in square boxes (**A**, **B**, **C**, **D**, **E**, and **β**). The parameter **A** denotes the likelihood mapping, i.e., the probability of making an observation o given state s. **B** Beliefs about how states transition into others. **C** Prior beliefs (preferences) about sensory outcomes. **D** Beliefs about the initial state before any observations. **E** Priors over policies—what the agent would normally do, independent of the EFE in the current situation. **β** Prior over gamma (γ), whereby γ—i.e., expected action precision weight—is the inverse of **β**. This generative model evinces hierarchical depth, in so far as the orange level (L2) contains higher-level states which constrain the state and policy priors at the level below (L1). These higher-level states are informed by the policy and preference priors that “belong” to a higher-order self that is enculturated and capable of being reflected upon. With respect to OPSM in TS, these parameters guide active tic suppression. However, they do not determine the beliefs at the lower (blue level). Here, the policy and preference priors—that shape action—are geared towards the suppression of interoceptive free energy (i.e., prediction errors). The agent makes the implicit inference by inverting their generative model: the hidden state that best explains observed outcomes is inferred, based on initial state beliefs (**D**), a likelihood mapping (**A**)—which, in this case, has extremely high precision weighting—and beliefs about state transitions (**B**). Although tics serve to suppress free energy at this lower-level, sensorimotor (blue) level, when their sensory outcomes associated are observed at the (orange) higher-level, free energy increases given that level’s architecture

A thorough analysis of the MPS and the validity of grounding it in interoceptive inference is beyond the scope of this paper (Christoff et al., 2011; Legrand, 2006, 2007; Legrand & Ruby, 2009; Limanowski & Blankenburg, 2013); nevertheless, if interoceptive prediction error suppression, which ticcing achieves, underwrites the MPS—even if this just means that they are necessarily involved in “our intuitive sense of being ourselves located where the body is felt and represented” (Legrand & Ruby, 2009, p. 272)—then the proposed conflict at hand becomes clear. Our point is that, in ticcing, the individual can be described as retaining the integrity not only of its own body but also its MPS. However, in ticcing, she is denying evidence for more explicit hypotheses about the type of agent that she is: that is, a non-ticcing thing. We term this *oppositional phenomenal self-modelling* (OPSM) (Fig. 2).

 This is not to say that ticcing leads to the disappearance of the self-concept, for a veridical self-concept is always available to the individual. Rather, it just acts as evidence against the preferences associated with that individual’s desired self-concept. Faced with this dialectic, she can either update her model and conclude that she is not a socially adaptive being, suppress her tics, or continue to tic, reinforcing the duality of desires and the prediction error therein. A thorough discussion of which of these options best serves the wellbeing of those with TS is beyond the scope of this paper; that said, we recognise that behavioural therapy for TS involves exposure and response prevention (ERP) and habit-reversal training (HRT) via tic suppression (Fründt et al., 2017; McGuire et al., 2015; Verdellen et al., 2007, 2011) and propose that the function of these interventions can be cast as the weakening of habitual ticcing behaviour, which thereby affords evidence for the (desired) presence of a non-ticcing, higher-order self(-concept) and thus increases a global sense of wellbeing. However, clearly more empirical work—informed by phenomenology—is necessary to disentangle the numerous forms of self-experience that are altered as a consequence of TS and the management of tics across development.

## Empirical Predictions, Limitations, and Future Directions

Although this paper is fundamentally theoretical, it is worth recognising that our model makes testable predictions worth exploring. In Section 1, we claimed that TS is marked by hyperprecision weighting over (the likelihood of) interoceptive sensory data. This is strongly consonant with the findings of Rae et al. (2019b), who found that participants with TS had an objectively lower interoceptive accuracy on a heartbeat tracking task, as well as an increased (self-reported) sensitivity to interoceptive signals (Ganos et al., 2015; Narapareddy et al., 2022). However, these researchers did not investigate the mechanism underlying tics and the subsequent attenuation of the premonitory sensation.

We have proposed that two processes might be at play. First, tics might engender a direct (mechanical) alteration of the interoceptive and proprioceptive state of the individual, as if scratching an itch. Second, tics entail an indirect down-weighting of the interoceptive prediction errors, because this form of sensory attenuation is necessary for movement along the proprioceptive arc. The degree to which one process predominates over another is testable: one would expect that if the occurrent sensory attenuation was solely due to the down-weighting of interoceptive prediction errors necessary to allow for movement, there would be minimal change in the *underlying state* of the epithelial receptors following the tic, and, rather, a change only in the precision weighting of sensory afferent signals.

Furthermore, given the causal role of insula reactivity within our model of tics and premonitory sensations, we propose that the validity of our account can be empirically tested with noninvasive methods such as low-intensity focused ultrasound (LIFU) (Bystritsky et al., 2011). Importantly, LIFU can be used to modulate neuronal activity in deeper areas of the brain like the insula. One prediction that our model makes is that direct inhibition of both the posterior and anterior insula will result in the transient suppression of tics (compared with sham), as shown for other interoceptive, homeostatic processes, including both the reaction to and perception of pain (Legon et al., 2023).

With respect to Section 2, we argue that sensation-free ticcing constitutes a form of epistemic foraging in the pursuit of evidence for one’s own agency (Perrykkad et al., 2023). To this end, we expect that individuals with TS may express tics as a form of exploratory behaviour that is driven by the opportunity to learn self-referential information. This could be tested via a multiarm bandit task within which participants could either opt for reliable, nonself-referential information in an exploitative fashion or pursue a riskier “arm,” which could offer self-referential information or none at all. This is in line with findings that receiving information is inherently rewarding (Bromberg-Martin & Hikosaka, 2009; Bromberg-Martin & Monosov, 2020; Charpentier et al., 2018; FitzGibbon et al., 2020; Garvin & Krishnan, 2022). Similarly, we would expect that, in the paradigm used by Perrykkad et al. (2023), participants with TS would spend more time foraging in environments that yield accurate beliefs about their own agency than a control group and would be more sensitive to an increase of prediction error regarding the state of their own agentive control. We therefore predict that TS participants would switch environments in the pursuit of greater certainty earlier than control groups, given the steady accumulation of uncertainty about agency in their current environment (Cohen et al., 2007, p. 937).

Finally, we suggested in Section 4 that TS likely engenders a type of OPSM, marked by a clash between the preferences associated with a higher-order, socially embedded, desired self-concept and those relating to lower-order sensorimotor arcs. Given the abstract nature of this account of ticcing behaviour and selfhood, we appeal to descriptions of TS phenomenology. We expect discernable differences in specific phenomenological aspects of the lived experience of those with TS and those without. More precisely, one hypothesis is that those with TS experience a lack of *self-coherence—*i.e., they often feel attracted by different desires: in this case, to tic or not to tic. This may have downstream effects on the sense of agency in TS. These experiential distinctions should be carefully studied using novel questionnaires, as well as (micro) phenomenological interviews (Bevan, 2014; Petitmengin et al., 2019; Valenzuela-Moguillansky & Vásquez-Rosati, 2019). It is worth stressing that the dynamics underpinning OPSM are likely at play in other psychopathologies; thus, the predictions made above should be transferable. For example, ERP also is the first-line psychotherapy for obsessive compulsive disorder (OCD) (Foa & Goldstein, 1978; Foa & McLean, 2016; Marks et al., 1975) and involves the provocation of anxiety-producing obsessions and the inhibition of compulsions that, whilst alleviating short-term anxiety, reinforce the individual’s fear (Hezel & Simpson, 2019). Thus, like TS, OCD can be described from the perspective of a duality of desires, which maps onto distinct temporal horizons: to tic/compulse (i.e., short-term alleviation of premonitory sensation/anxiety) versus to not-tic/not-compulse (i.e., long-term reinforcement of desired conceptual self-model). This, again, could be tested via questionnaires and phenomenological interviews.

In fact, we suggest that our four-tiered model may provide explanatory power beyond TS and OCD, especially because it is unclear to what degree tics (or at least tic-like movements) are exclusive to TS. Indeed, certain aspects of our model might be able to generate insights into the neurocognitive processes relevant to the expression of motoric phenomena that resemble tics within both hypo- and (especially) hyper-kinesias, including self-stimming in autism spectrum disorder ASD (Nwaordu & Charlton, 2023) but also stimulus utilisation behaviours (Janik et al., 2018), functional tics (Cavanna et al., 2023), and attention deficit hyperactivity disorder (ADHD). For example, in ASD, individuals may produce self-initiated (rhythmic) actions to regulate sensory input (e.g., to ameliorate symptoms of hypersensitivity), otherwise known as self-stimming. We propose that these movements may be aptly described within the lower-level sensorimotor aspects of our model, which provides a general mechanism by which to reduce sensorimotor prediction error through movement. Furthermore, qualitative work by Kapp et al. (2019) highlights that autistic individuals perceive self-stimming to be generally socially unacceptable and yet capable of being understood if explained to others, which suggests that there is likely an element of OPSM at play among self-stimming individuals, which nevertheless can be tempered or moderated given social acceptance. However, whilst the present model may in fact shed light upon the expression of such behaviours at a conceptual level, its generalisation to other conditions, such as ASD, also must be empirically tested. A key challenge to our account is therefore to disambiguate TS specific phenomena from non-TS specific phenomena and assess the degree to which our model has explanatory power beyond the purview of TS.

It is worth noting some of the limitations of this paper and the model it proposes. One such limitation of our model is that it does not make concrete predictions about when one preference within the OPSM dialectic might predominate over the other. As mentioned, however, the behavioural therapy that is typically employed for the management of TS could be described as weakening the prior value—encoded in the E tensor—of tics, whilst strengthening the prior value of tic suppression. With respect to OPSM, this can be cast as biasing the duality of desires towards the (non-ticcing) states consequent on the actions associated with the desired self-concept (the non-ticcing agent), in spite of the felt discomfort that accompanies tic suppression. In other words, one would expect that the explicit preferences associated with the higher-order self-concept (i.e., tic suppression) will dominate action selection in those individuals with TS who have undergone ERP or HRT compared to those who have not.

In terms of other limitations, it is worth mentioning that the ambitious scope of our model should be tempered with rigorous empirical tests of our key hypotheses. As noted, we provide an analysis of four levels of ticcing behaviour and suggest specific predictions that can be used to assess the overall viability of our model. Importantly, however, we do not intend to provide a unitary explanation of ticcing behaviour, but instead aim to account for greater variability in ticcing behaviour in TS. For example, we suggest that the insula cortex plays a causal role in the generation and expression of tics, such that the transient inhibition of both anterior and posterior insula will result in the cessation of tics. However, the development of ticcing behaviour in the first place is an issue that we do not address, and it is likely that other neurocognitive processes contribute to the emergence (and maintenance) of such actions.

Another future challenge to address with our model is whether it can better account for observable behaviour over and above current models, including reinforcement learning (RL)-based approaches, which have demonstrated considerable success in characterising the computational basis of exploratory behaviour, which, in Section 2, we suggested underlies sensation-free ticcing (Alejandro & Holroyd, 2024; although see Friston et al., 2009; Tschantz et al., 2020). To this end, further evidence through model comparison is needed and can be applied to specific levels of analysis to evaluate the relative strengths and weaknesses of our characterisation of ticcing behaviour. However, we need more data; despite a steady increase in the public awareness of aspects of TS, there is still a noticeable absence of research programmes that can address many of the issues in TS mentioned in this work. Our hope is to motivate further experimental research in these directions and, alongside open and data-driven methods, promote a more holistic understanding of TS.

## Conclusions

We have provided a formal account of the symptomatology of TS through the lens of active inference and hierarchical generative modelling, offering a novel perspective on the interplay between sensorimotor, cognitive, and phenomenological aspects of the condition. We have offered a fourfold analysis of the cardinal features of TS, including ticcing behaviour and its control and variable complexity. We first focused on simple forms of ticcing behaviour aimed at reinstating sensory attenuation, which we theorise is driven by an episodic failure to attenuate sensory precision weight (that engenders premonitory sensations). We then analysed an epistemic variety of tics—a form of epistemic foraging—that occur in the absence of atypical sensation and described how these tics provide evidence for the organism’s world model and their status as agentive selves therein. We then characterised both epistemic (sensation-free) and nonepistemic (sensational) tics as habitualised behaviour, cast as strong prior beliefs about action selection. Finally, we show how such dynamics plausibly underscore a conflict in inference over the minimal phenomenal sense of self and a conceptual self-construction. The form of OPSM that we have proposed sheds light on the duality of desires experienced by individuals with TS, navigating between the urge to tic for homeostatic restoration and the imperative to suppress tics within the social environment. This formal account not only advances our understanding of TS but also invites further empirical and theoretical exploration into the intricate dynamics of self-experience and adaptive behavioural control in psychopathology more broadly, whose various manifestations might be defined, to a lesser or greater degree, by the mechanisms underwriting OPSM.

## Data Availability

The use, distribution or reproduction in other forums is permitted, provided the original author(s) or licensor are credited and that the original publication in this journal is cited, in accordance with accepted academic practice. No use, distribution or reproduction is permitted which does not comply with these terms.

## Footnotes

1 For a summary of the controversy surrounding the pathophysiological basis of TS, see Singer and Augustine (2019).

2 Note that this is the predominant model when accounting for behaviour in continuous state spaces. When discrete variables are considered, such as when decisions are made, the requisite hierarchical generative model frequently takes the form of partially observable Markov decision processes (POMDPs) (Da Costa et al., 2023; Friston et al., 2017b).

3 Note that, in this treatment, for simplicity, we are equating the next action (*u*) with policy. When a policy entails a sequence of actions, it is usually denoted by *π* in the active inference literature.

4 The question of whether active inference can account for so-called pre-reflective self-awareness - i.e., the “for-me-ness” immanent (and yet not objectified) in experience - is beyond the scope of this paper and ought to be explored elsewhere (cf., Parvizi-Wayne et al., 2024). This is not something that Hohwy and Michael (2017) consider: indeed, they argue that “the self is identical to these causes” (p. 8), without justifying how this gives rise to subjectivity and not (merely) the sense of self-as-object, to which certain features or contents are attributed (cf., Hohwy & Michael, 2017, p. 27; Legrand & Ruby, 2009). However, this is also not the critical issue on which this paper rests.

5 Although it is worth noting that, in many cases, those with TS advocate for wider acceptance within society, and make a deliberate choice not to manage their tics through medication.
